# Supplementary material for: The replication initiation determinant protein (RepID) modulates replication by recruiting CUL4 to chromatin
Source: Nat Commun. 2018 Jul 17;9:2782. doi: 10.1038/s41467-018-05177-6 (PMC6050238; doi:10.1038/s41467-018-05177-6)
Supplement: Supplementary file 1 — Supplementary Information [file 41467_2018_5177_MOESM1_ESM.pdf]

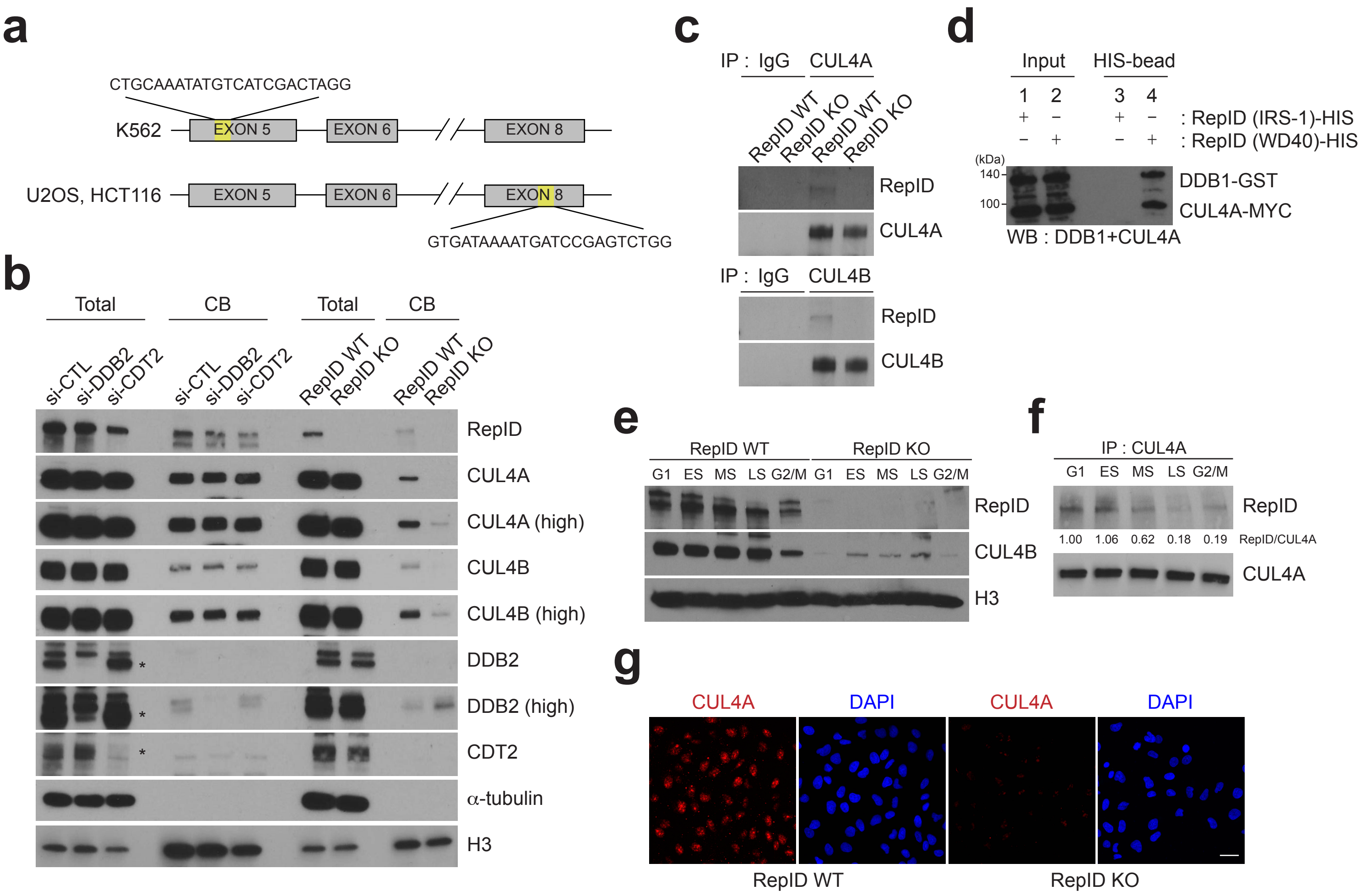

Supplementary Figure 1 (a) Schematic representing RepID KO generation by the CRISPR-Cas9 gene editing system in K562, HCT116 and U2OS. Target sequences are indicated in exon 5 for K562 cells and in exon 8 for U2OS and HCT116 cells. **(b)** U2OS WT cells were transfected with siRNA-DDB2 or siRNA-CDT2 and immunoblot analysis was performed to measure levels of chromatin-bound CUL4. RepID WT and RepID KO cell lysates were used as positive and negative controls. Asterisks indicate DDB2 or CDT2 signals. Histone H3 and α-tubulin were used as markers for cell fractionation. **(c)** Endogenous binding between RepID and CUL4. Chromatin fractions from U2OS RepID WT and RepID KO cells were immunoprecipitated using CUL4A or CUL4B antibodies and co-precipitated RepID was analyzed by immunoblotting. **(d)** In vitro binding between RepID and CRL4 using tagged purified proteins. **(e)** RepID WT and KO K562 cells were fractionated into cell cycle phases by elutriation and chromatin-bound CUL4B was analyzed by immunoblotting. ES, early S; MS, middle S; LS, late S. **(f)** Chromatin fractions from elutriated K562 RepID WT cells were immunoprecipitated using CUL4A antibody and co-precipitated RepID was detected by immunoblotting. **(g)** Endogenous chromatin-bound CUL4A detected by CUL4A-specific antibodies (ab92554) in U2OS RepID WT and KO cells after pre-extraction of soluble proteins (Color threshold: minimum 200, maximum 65,535). Scale bar indicates 25 μm.

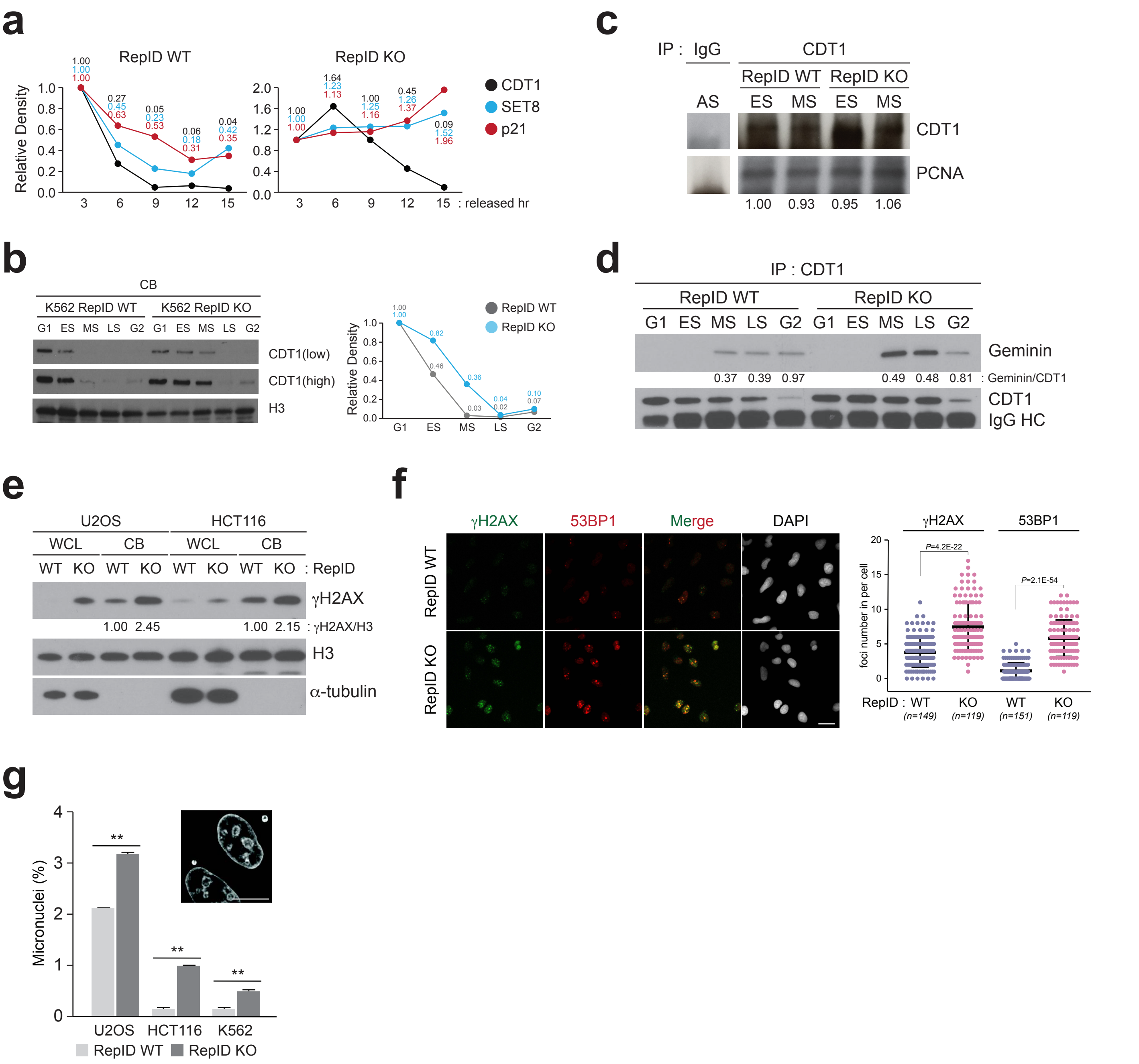

Supplementary Figure 2 (a) Quantification of CDT1, SET8 and p21 from nocodozole arrested, mitotic RepID WT and RepID KO HCT116 cells released in fresh medium and collected every 3 hours. Relative densities of western blot protein signals were normalized using the 3 hr time point (data related to Fig. 2e). **(b)** Chromatin-bound CDT1 levels in elutriated WT and KO K562 cells. Graph showing the relative CDT1 densities normalized using CDT1 signals from G1 is shown on the right. **(c)** Fractionated RepID WT and KO K562 cells were used for CDT1 immunoprecipitation in chromatin-bound fractions of ES and MS. Co-precipitated PCNA was analyzed by immunoblotting and the ratios PCNA/CDT1 are indicated. Asynchronized cells and rabbit-IgG were used as controls. **(d)** CDT1 immunoprecipitation using soluble nucleuar and chromatin-bound extracts from fractionated RepID WT and KO K562 cells. Co-precipitated geminin was analyzed using immunoblotting and the ratios Geminin/CDT1 calculated. **(e)** γH2AX levels on chromatin in RepID WT or KO cells. **(f)** γH2AX and 53BP1 foci per cell in U2OS RepID WT and KO cells (Color threshold: minimum 200, maximum 40,000). Scale bar indicates 20 μm. **(g)** Percentage of cells with micronuclei in RepID WT and RepID KO cells in HCT116, U2OS and K562. Ten thousand cells were counted per sample. A representative image of micronuclei is shown. Bar graphs = standard errors. **P < 0.01.

a

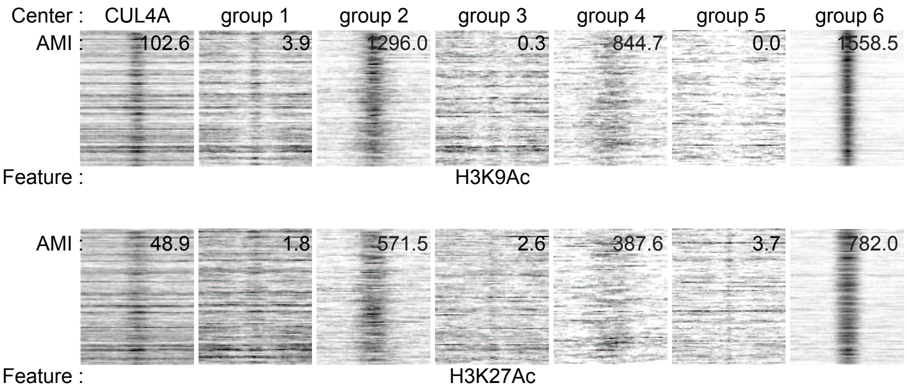

b

Total peaks		Associated with early origins 71,391 peaks		Associated with middle origins 17,360 peaks		Associated with late origins 36,973 peaks	
Sample	Peak number	Peak number	%	Peak number	%	Peak number	%
CUL4A	56,715	32,063	44.9	4,871	28.1	6,390	17.3
RepID	24,222	15,217	21.3	1,629	9.4	1,755	4.7
CDT2	28,516	12,582	17.6	2,477	14.3	4,190	11.3

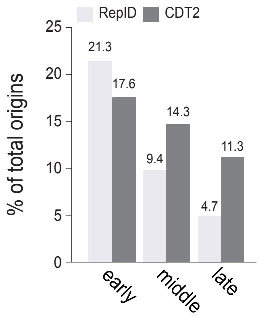

Supplementary Figure 3 (a) Heat map depicting the colocalization between the acetylated histone H3 (H3K9Ac or H3K27Ac) and the ChIP-seq peaks of CUL4A and the peaks of the six subgroups described in Fig. 3b. **(b)** Extent of colocalization between early, mid and late replication origins with ChIP-seq peaks from CUL4A, RepID and CDT2 (table, left panel; histogram, right panel).

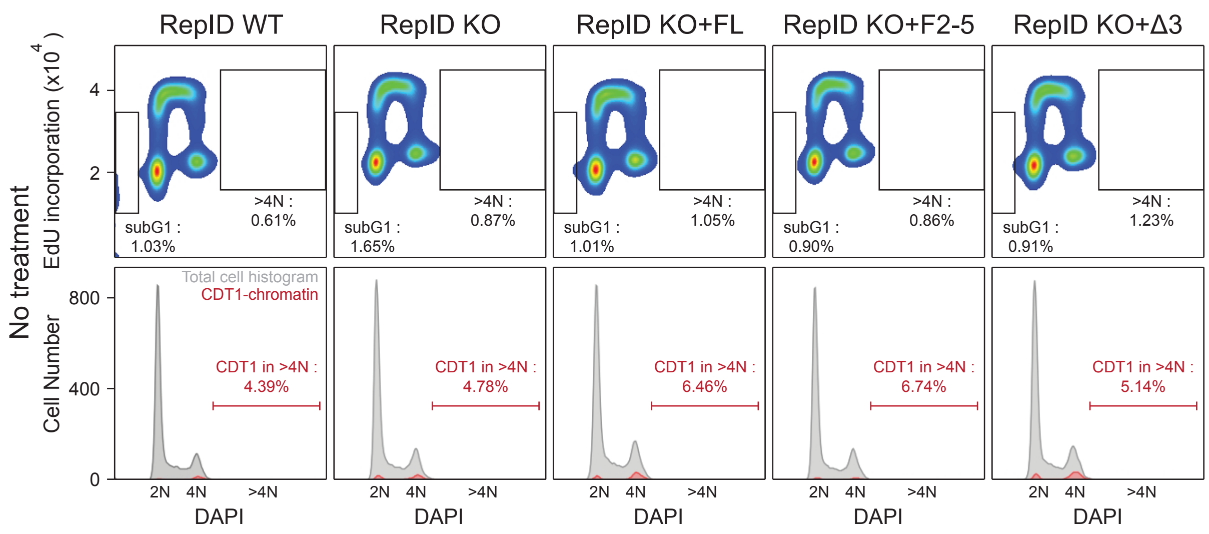

Supplementary Figure 4 Untreated WT, RepID KO, or RepID KO+RepID fragment-expressing U2OS cells were labelled with EdU for 30 min and analyzed by FACS. Percentages of re-replicating and SubG1 cells are indicated. Chromatin-bound CDT1 distribution throughout the cell cycle was analyzed by FACS using an antibody directed against CDT1 after pre-extraction of soluble nuclear proteins. Total cell histogram and CDT1-bound chromatin histogram are shown, and percentages of chromatin-bound CDT1 in re-replicating cells are indicated in red (bottom panel). This figure is the control for the Fig. 4d and 5g.

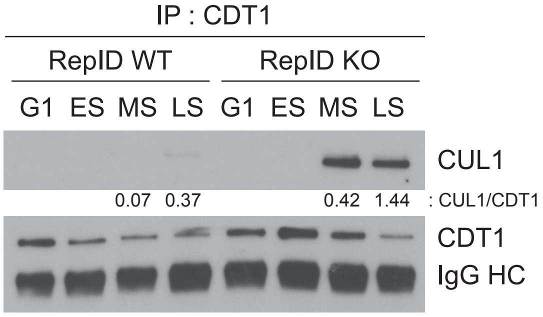

Supplementary Figure 5 Interactions between CDT1 and CUL1 are increased in RepID KO cells during middle and late S phase. Soluble nucleuar and chromatin-bound fractions from K562 WT and KO RepID cells were used for CDT1 immunoprecipitation. Co-precipitated CDT1 and CUL1 were analyzed by immunoblotting. Numbers represent ratios for CUL1 and CDT1.

Fig. 1a

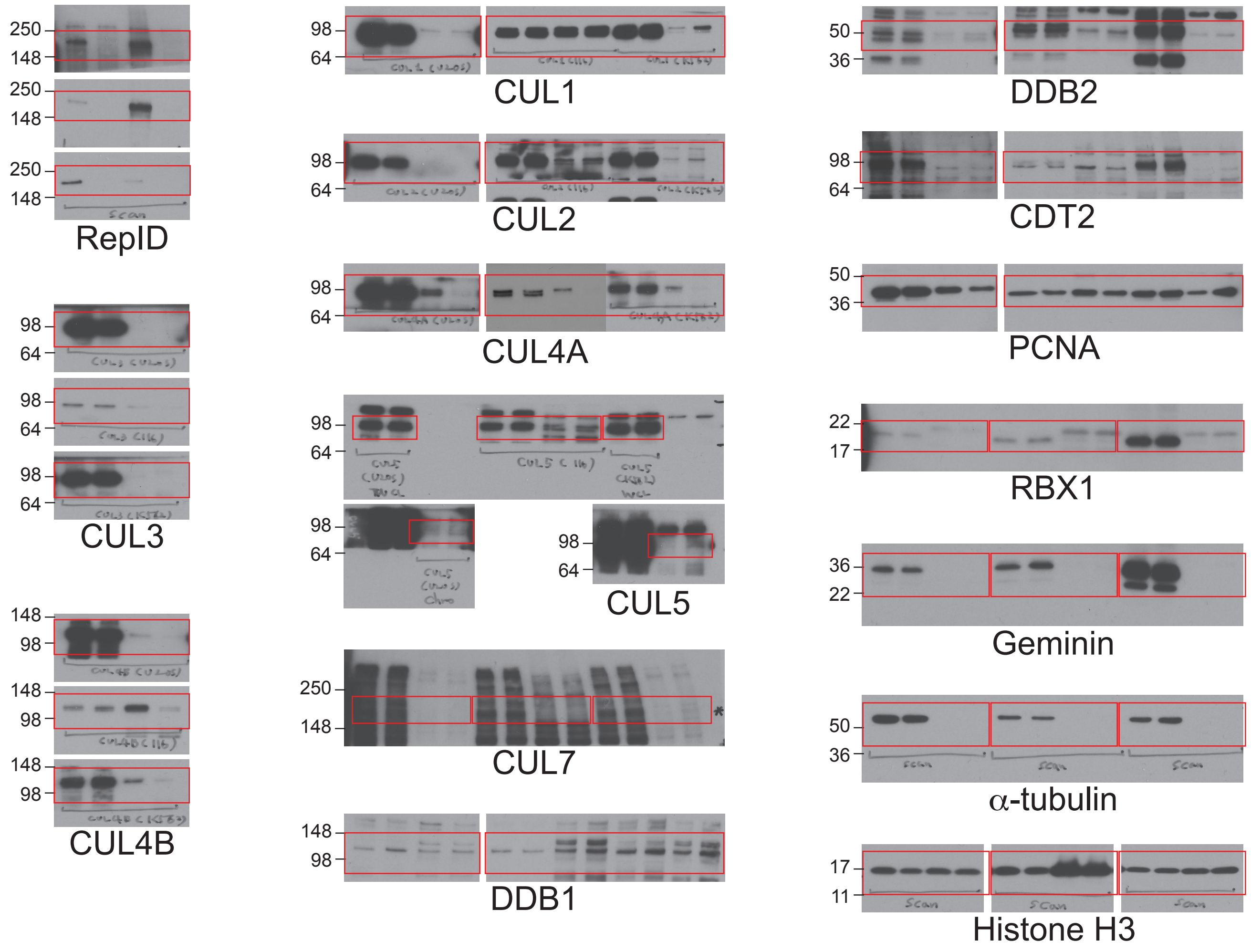
RepID
250
148
CUL3
98
64
CUL4B
148
98
CUL1
CUL2
CUL4A
CUL5
CUL7
250
148
DDB1
DDB2
50
36
CDT2
PCNA
RBX1
22
17
Geminin
α-tubulin
Histone H3
11
*

Fig. 1d Fig. 1e Fig. 1g

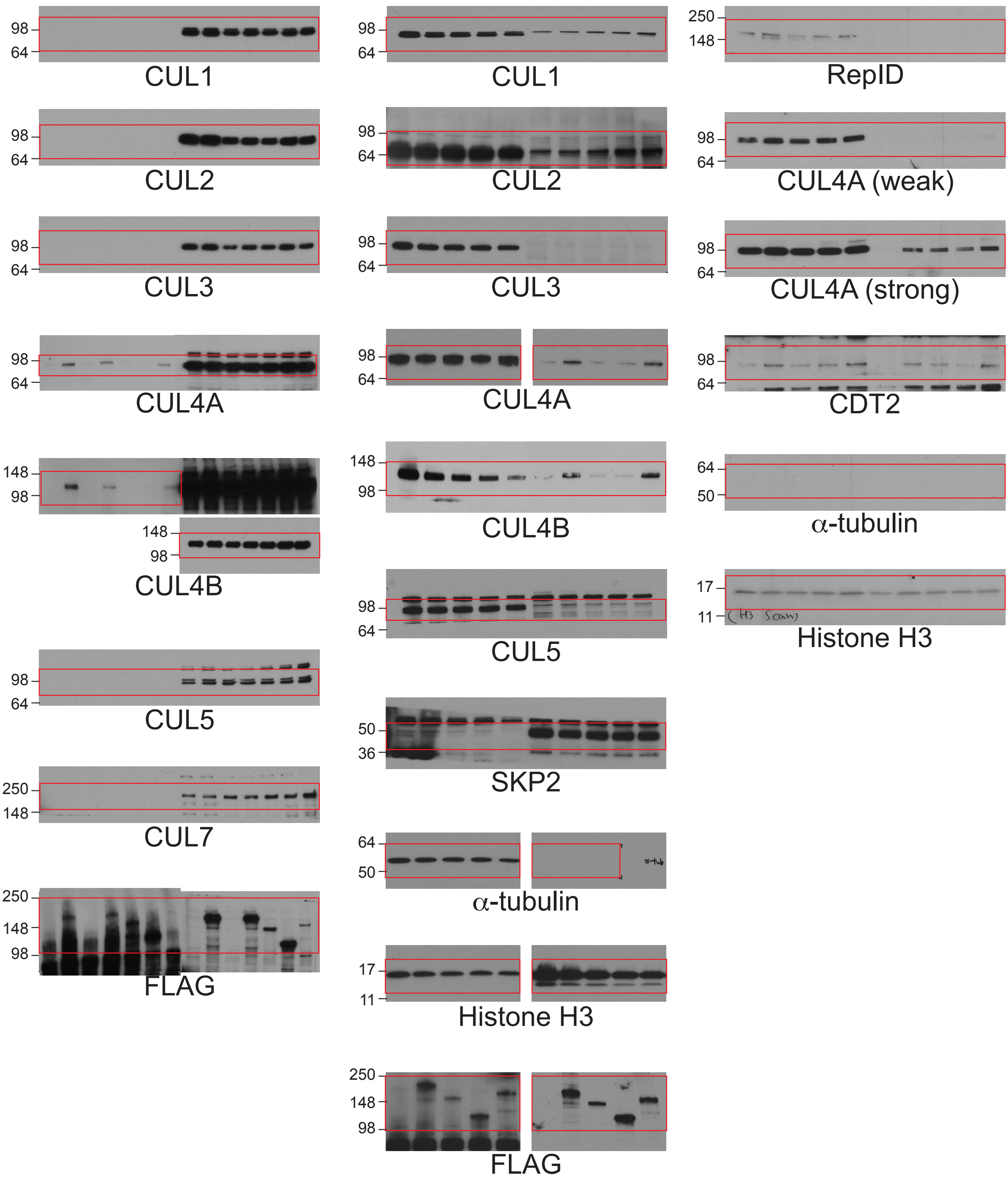

Fig. 2b

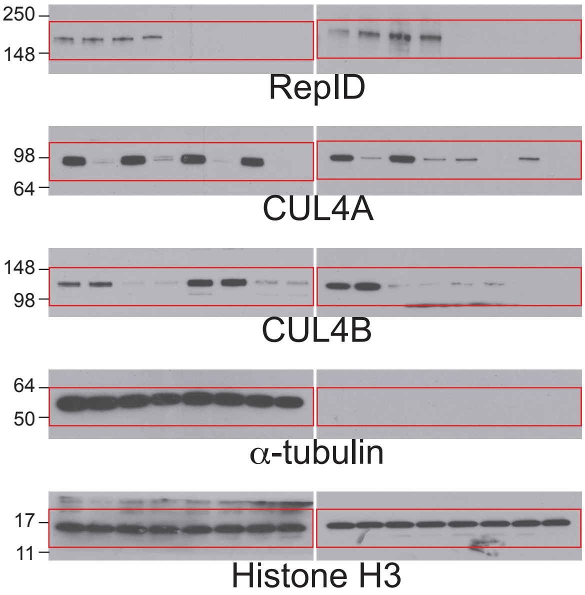

Fig. 2e

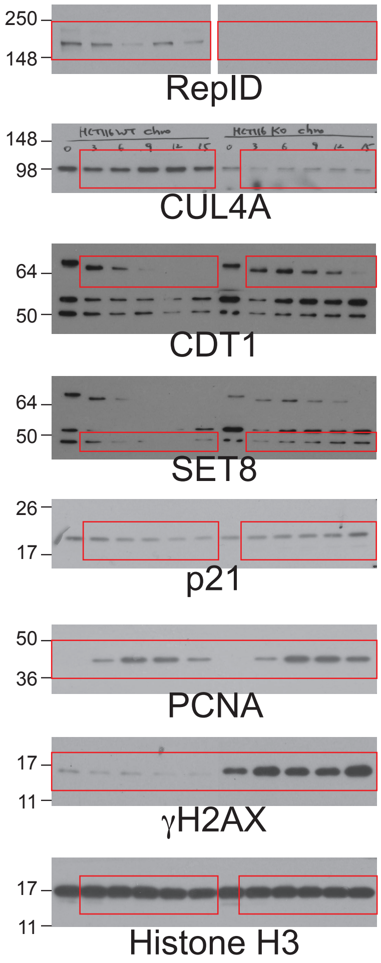

Fig. 4c

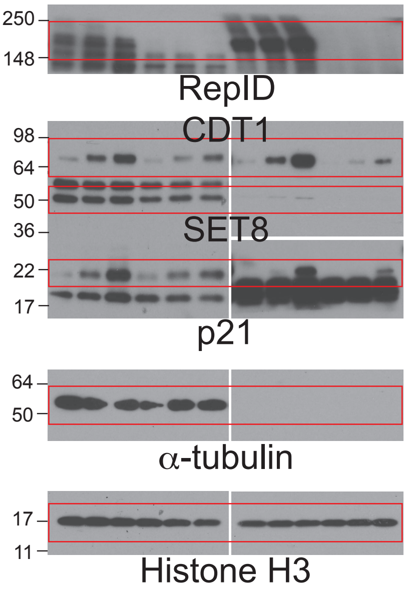

Fig. 4e

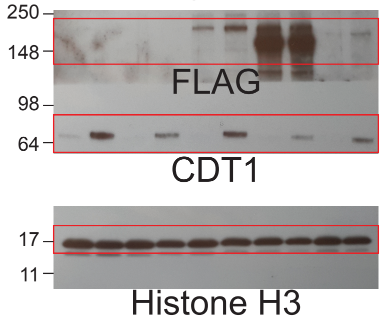

Fig. 5a

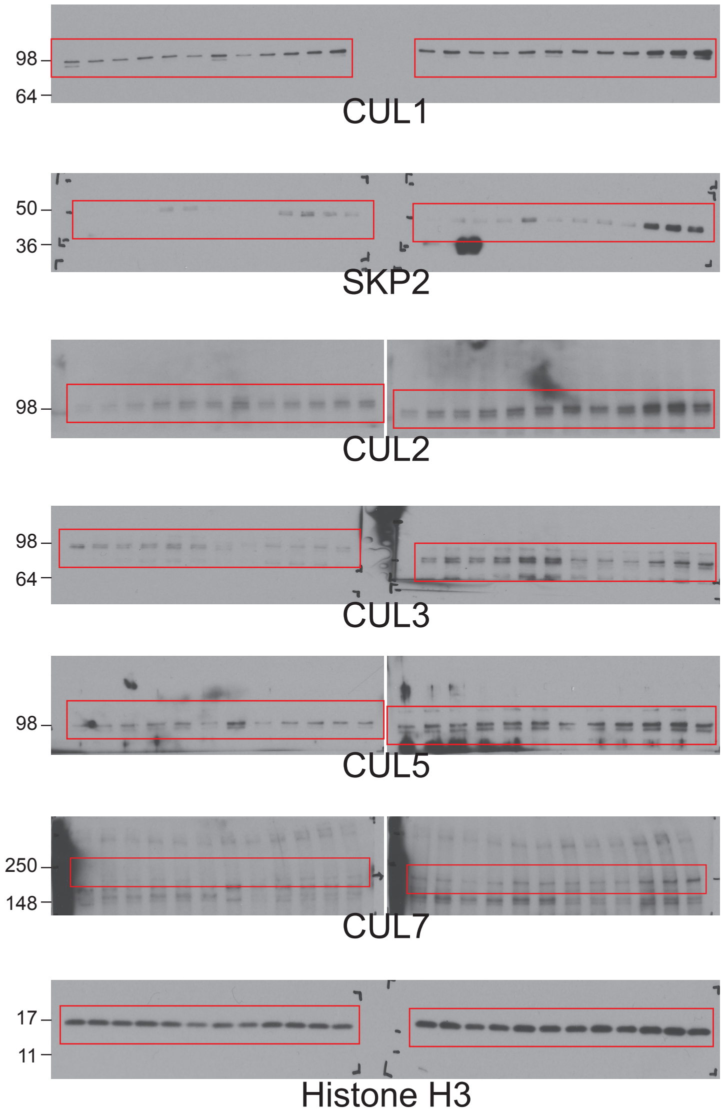
98
64
CUL1
50
36
SKP2
98
CUL2
98
64
CUL3
98
CUL5
250
148
CUL7
17
11
Histone H3

Fig. 5h

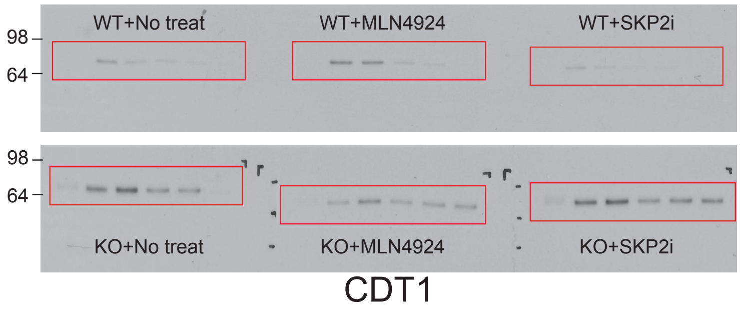
WT+No treat
WT+MLN4924
WT+SKP2i
98
64
98
64
KO+No treat
KO+MLN4924
KO+SKP2i
CDT1

Supplementary Fig. 1b

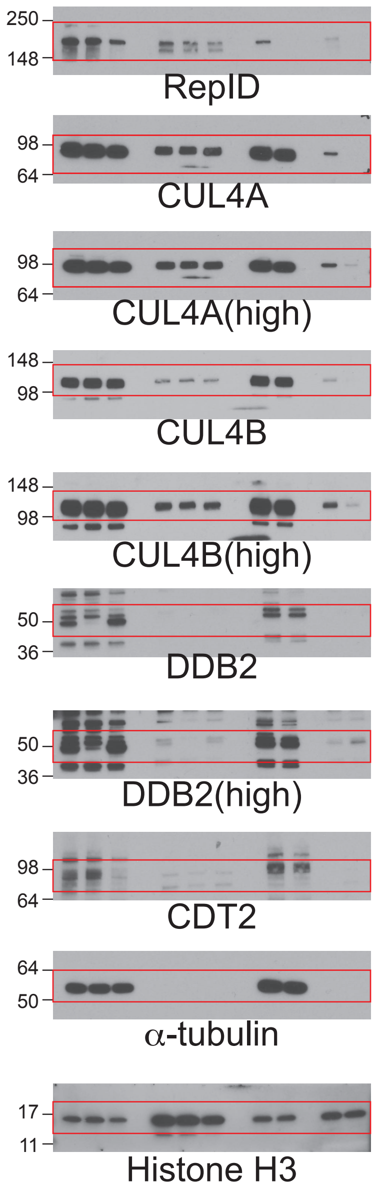

Supplementary Fig. 1c

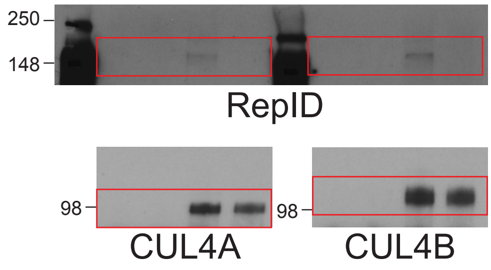

Supplementary Fig. 1d

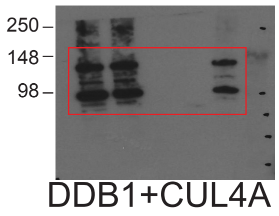

Supplementary Fig. 1e

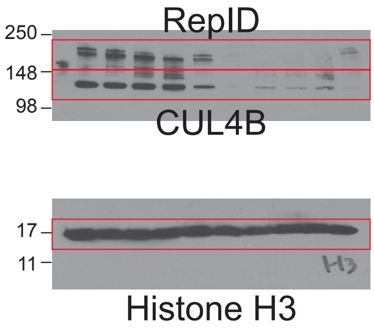

Supplementary Fig. 1f

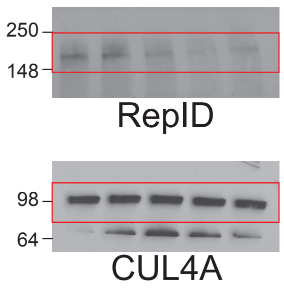

Supplementary Fig. 2b

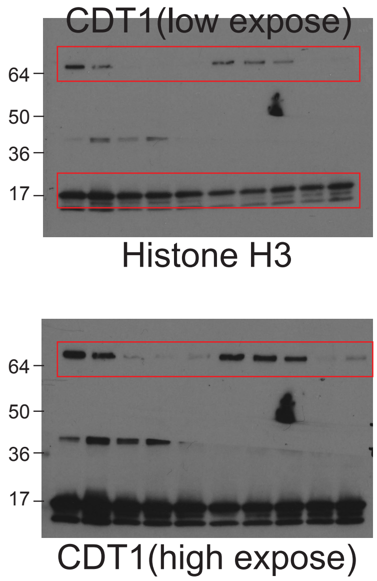

Supplementary Fig. 2c

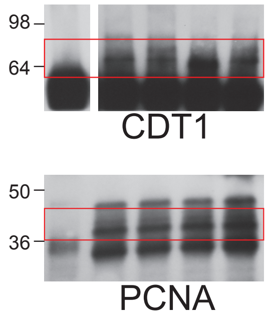

Supplementary Fig. 2d

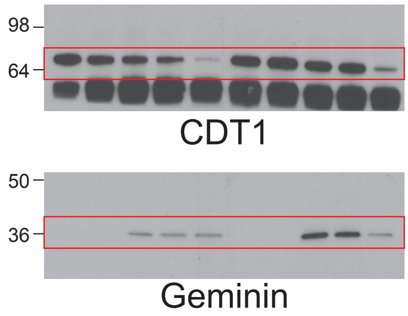

Supplementary Fig. 2e

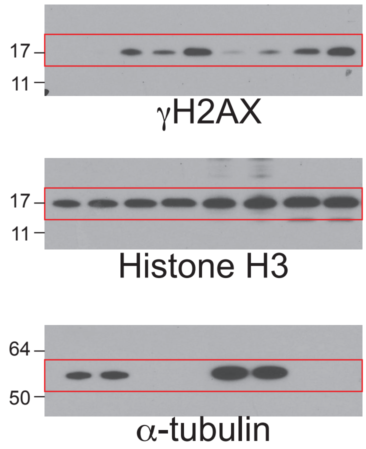

Supplementary Fig. 5

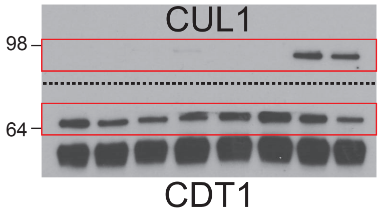

Supplementary Figure 6 Unprocessed original scan files for immunoblot analysis.
