# Supplementary material for: The replication initiation determinant protein (RepID) modulates replication by recruiting CUL4 to chromatin
Source: Nat Commun. 2018 Jul 17;9:2782. doi: 10.1038/s41467-018-05177-6 (PMC6050238; doi:10.1038/s41467-018-05177-6)
Supplement: Supplementary file 3 — Description of Additional Supplementary Files [file 41467_2018_5177_MOESM3_ESM.pdf]

Description of Additional Supplementary Files

File Name: Supplementary Data 1
Description: Quantification and statistical analysis of data collected from immunofluorescence microscopy. This data is related to Fig. 1h and Fig. 2c.
